# Improving ambulance care for children suffering acute pain: a qualitative interview study

**DOI:** 10.1186/s12873-022-00648-y

**Published:** 2022-06-03

**Authors:** Gregory Adam Whitley, Pippa Hemingway, Graham Richard Law, Aloysius Niroshan Siriwardena

**Affiliations:** 1 Community and Health Research Unit, Sarah Swift Building, School of Health and Social Care, University of Lincoln, Brayford Wharf East, Lincoln, England LN5 7AT UK; 2 Clinical Audit and Research Unit, East Midlands Ambulance Service NHS Trust, Lincoln, England UK; 3 Faculty of Medicine and Health Sciences, University of Nottingham, Nottingham, England UK; 4 Community and Health Research Unit, Lincoln Medical School, University of Lincoln, Lincoln, England UK

**Keywords:** Adolescent, Analgesics, Child, Emergency medical services, Pain

## Abstract

**Background:**

Pain is a highly complex sensory and emotional experience. When a child suffers acute pain through illness or injury, they are often transported to hospital by ambulance. Pre-hospital pain management in children is poor, with 61% of children receiving suboptimal pain management. Consequences of poor pain management include the risk of developing post-traumatic stress disorder and altered pain perception. We aimed to identify clinicians’ perceptions of barriers, facilitators and potential improvements for the management of pre-hospital acute pain in children.

**Methods:**

Qualitative face to face semi-structured recorded interviews were performed in one large UK ambulance service. Audio files were transcribed verbatim with thematic analysis used to generate themes. NVivo 12 was used to support data analysis. Findings were combined with existing evidence to generate a driver diagram.

**Results:**

Twelve ambulance clinicians participated, including 9 registered paramedics and 3 emergency medical technicians. Median (IQR) age was 43.50 (41.50, 45.75) years, 58% were male, median (IQR) experience was 12 (4.25, 15.50) years and 58% were parents. Several themes relating to barriers and facilitators were identified, including physical, emotional, social, organisational, environmental, management, knowledge and experience. Improvement themes were identified relating to management, organisation and education. These data were combined to create a driver diagram; the three primary drivers were 1) explore methods to increase rates of analgesic administration, including utilising intranasal or inhaled routes; 2) reduce fear and anxiety in children, by using child friendly uniform, additional non-pharmacological techniques and more public interaction and 3) reduce fear and anxiety in clinicians, by enhancing training and optimising crew mix.

**Conclusions:**

The quality of care that children receive for acute pain in the ambulance service may be improved by increasing rates of analgesic administration and reducing the fear and anxiety experienced by children and clinicians. Future research involving children and parents would be useful to determine the most important outcome measures and facilitate intervention development.

**Supplementary Information:**

The online version contains supplementary material available at 10.1186/s12873-022-00648-y.

## Background

During 2019–20 in England, over 430,000 children under 18 years of age were transported to accident and emergency departments by ambulance, with those aged under 5 years constituting the largest proportion (60%) [1]. Approximately 20% of children attended by ambulance suffer acute pain [2]. Pain is ‘an unpleasant sensory and emotional experience associated with, or resembling that associated with, actual or potential tissue damage’ [3]. Access to pain management is considered a fundamental human right [4] and effective pain management has recently been identified as a key quality outcome measure for ambulance services [5]. Despite this, pre-hospital pain management in children is considered poor, [6, 7] with only 39% of children achieving effective pain management [2]. Without effective pain treatment, children are at risk of adverse consequences including post-traumatic stress disorder [8, 9] and altered pain perception [10, 11].

A recent systematic mixed studies review identified and synthesised five qualitative studies assessing the barriers and facilitators to pre-hospital pain management in children [12]. There were no studies that explored the experiences of children whilst only one study explored the experiences of parents during the ambulance encounter [13]. Parents felt that alleviation of their child’s pain was important, as was a family-centred approach that included parents in the management [13]. Since the review, a UK study reported barriers and facilitators to pain assessment and management in the UK and concluded that situational, organisational and personal factors influence paramedic management of traumatically injured children [14].

The aim of this study was to explore barriers, facilitators and potential improvements for pre-hospital acute pain management in children, from the perspective of UK ambulance clinicians (paramedics and emergency medical technicians (EMTs)).

## Methods

### Study design and setting

A generic qualitative study [15] was performed in a large regional UK ambulance service: the East Midlands Ambulance Service NHS Trust (EMAS). This study report has two objectives; 1) identify barriers and facilitators to the pre-hospital pain management process in children and 2) explore potential methods of improvement. This paper reports recommendations for future research, policy and clinical practice improvement.

EMAS is one of 10 ambulance services in England and serves the counties of Nottinghamshire, Derbyshire, Leicestershire, Rutland, Lincolnshire and Northamptonshire. It serves a population of 4.8 million, including an estimated 996,348 children (21%) under the age of 18 years [16]. It covers an area of 16,710 km^2^ covering both urban and rural areas [17]. Approximately 2215 emergency calls are received per day and EMAS employ over 4000 staff, of which over 2700 are ambulance staff [17].

### Sampling

All EMAS ambulance clinicians were invited to participate by email and service newsletter. Clinicians who expressed an interest were sent a participant information sheet, a privacy notice and had the opportunity to ask any questions before they were invited for interview.

Participants were selected purposively using maximum variation sampling [18]. Paramedics and EMTs were recruited; we included clinicians of both sex and with a range of clinical experience. Inclusion criteria were:

- Employed by EMAS as a paramedic, EMT or emergency care practitioner (paramedic with enhanced primary care skills).
- Working on active front line duties during the 12 month period prior to interview.

Sampling continued until data saturation was achieved; no new codes or meaning were gained from additional data [19]. Interviews were conducted from August to November 2019.

### Data collection

Data were collected from face-to-face semi-structured interviews via audio recordings on EMAS premises. Only the interviewer and interviewee were present during the session. An interview schedule was used as a prompt and to collect field notes (see Additional file 1); the development of the interview schedule was informed by our previous systematic review [12] and the first phase of our mixed methods study [2]. The interview schedule was pilot tested on the first three participants; no substantial changes were required. Each participant was asked to provide a vignette of a recent experience treating a child in pain as an ice-breaker to start the interview [20]. Interviews lasted approximately 60 minutes.

Written informed consent was gained from participants prior to the interview. Participants were anonymised by assigning a sequential number preceded by ‘P’ for paramedics and ‘T’ for EMTs; this labelling was necessary to explore reasons why children attended by paramedics were more likely to achieve effective pain management than those attended by EMTs [21]. Transcripts were not returned to participants for comment but were checked several times for accuracy by GAW.

### Reflexivity

Interviews were performed by GAW who was positioned within the critical realist framework [22]. As a paramedic and former EMT, GAW shared the culture and prior understanding of the clinical participants [23] enabling the pursuit of more in depth details, as simpler concepts and terminology did not require explanation. There was a minor concern that this may have created ‘blind spots’ [24] where seemingly simple concepts that are taken for granted may have been overlooked. At the time of the interviews, GAW was a full-time PhD student and a part-time paramedic. GAW had no formal qualitative training however he did receive informal training and advice from members of the Community and Health Research Unit; specifically, from Dr. Sirdifield and Mr. Phung. Participants were aware that GAW was a PhD student and a professional relationship was already established with some (*n* = 5) of the participants prior to interview. The interview schedule (see Additional file 1) helped to minimise the influence of GAW on data collection and neutral, short questions and follow up prompts were used.

### Data analysis

Audio recordings were transcribed verbatim by GAW. Thematic analysis [25] was used to analyse the data within QSR NVivo version 12. The steps of analysis included; 1) familiarisation with the data, 2) generating initial codes, 3) searching for themes, 4) reviewing themes, 5) defining and naming themes and 6) producing the report [25]. Coding was performed by GAW, with all authors involved in the discussion and iterative refining of codes and themes. Several iterations of the thematic maps were designed, each of which underwent rigorous review within the team and externally; this visualisation was fundamental to the grouping of themes and sub-themes and development of the final thematic structure.

The analysis of barriers and facilitators was considered both inductive (generation of initial codes, generation of some themes and sub-themes) and deductive (use of frameworks); the biopsychosocial model of health [26], comfort theory [27] and competency frameworks [28] were used to create a theoretical framework. The physical and environmental aspects of Comfort Theory were combined with the psychological (emotional) and social aspects of the biopsychosocial model along with the competencies of knowledge and experience from competency frameworks, with the addition of management and organisational factors to create a bespoke framework that was used after the initial coding. The analysis of proposed improvements was considered purely inductive; no theoretical framework was used.

Respondent validation was not performed as its ability to provide validity is questionable; a thorough analysis of qualitative data often involves navigating contradictions and conflicts between participants; participants are neither right nor wrong, but the conflict itself provides useful insights [18].

### Patient and public involvement

The research question and study design were informed through discussion with the Healthier Aging Patient and Public Involvement (HAPPI) group at the University of Lincoln. It was concluded that pre-hospital pain management in children was an important topic of research and that this study should help develop a more comprehensive understanding of the problem.

## Results

Overall, 25 clinicians expressed an interest and 12 participants were included in this study, see Additional file 1 for the summary of participant characteristics. Median (interquartile range (IQR)) age was 43.5 (41.5, 45.75) years, 58% were male, 75% were paramedics, median (IQR) experience was 12 (4.25, 15.5) years and 58% were parents.

### Barriers and facilitators

**Fig. 1:**
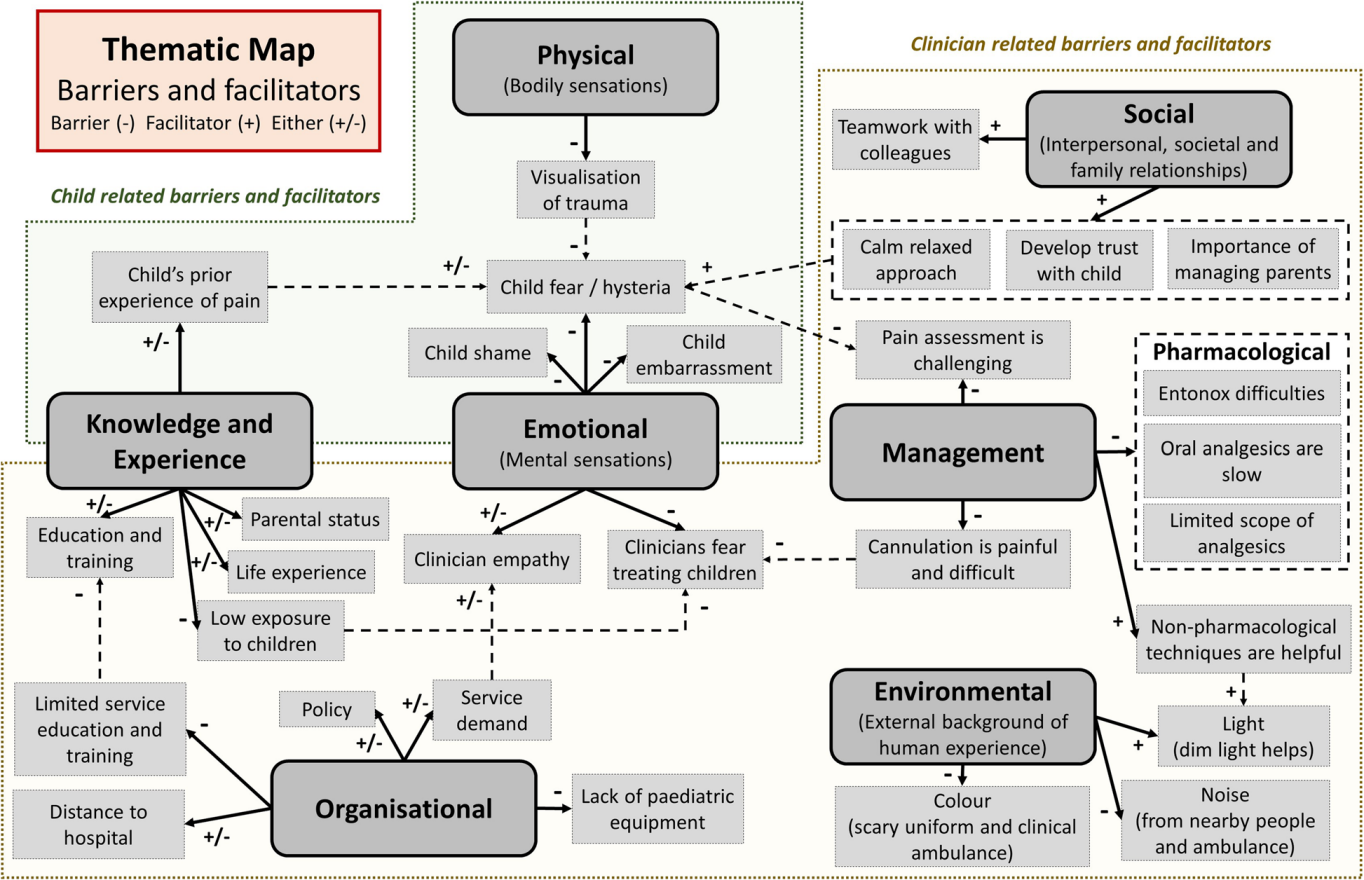
Thematic map of barriers and facilitators

A thematic map was generated to illustrate the barriers and facilitators, see Fig. 1. The thematic map was split into “child related” and “clinician related” barriers and facilitators. Although the child’s perspective was not directly assessed, it was felt the clinician’s perceptions and experiences provided valuable insights into child-related factors; clinicians attend children with and without acute pain and are well placed to compare these experiences.

Barriers and facilitators revolved around several major themes; physical, emotional, social, organisational, environmental, management along with knowledge and experience. These major themes were informed by sub-themes, which were informed by direct quotations, **see** Additional file 1 for the complete table of supporting quotations.

#### Supporting quotations

Participants acknowledged the influence of children’s emotions on the management of acute pain, as children often presented with fear and anxiety, which can be difficult to separate from pain:

*‘I think it’s very difficult to distinguish what is fear and what is pain, and we could end up highly scoring a child for pain, because they’re hysterical, and saying they’re a 10 out of 10 pain and we could end up over-treating … because maybe we’re treating fear’*

Participant P03

The physical visualisation of trauma was identified as a potential barrier; clinicians stated that as children notice a visual change to their body, fear and anxiety may be exacerbated:

*‘they can see that something has changed on their body as opposed to something that’s, inside the torso … That they can’t, that they have no idea what, what’s causing it. That, that could equally be as, as traumatic but, I think v-visualising something can be as bad if not worse … ’*

Participant P01

Participants stressed the importance of a calm and relaxed approach by a clinician and how the intensity of the situation can be reduced within a short period of time:

*‘Like you say, it’s a matter of calming them down isn’t it initially and, trying to get the, because when you go in, that whole situation is quite heightened isn’t it, but it’s very different after about 10 minutes when you’ve built up a bit of a rapport and you’ve got them a little bit quieter and calmer you can probably get a, a truer sense of, of what’s happening and how they’re feeling, as opposed to when you’re going in and they’re screaming and crying initially’*

Participant T03

Communication was also considered key, particularly body language and the clinician’s ability to manage the situation and build trust with the child:

*‘the interaction with the child, I think gaining the trust erm, is a massive thing, it, once you’ve lost that with a child, sometimes it doesn’t really matter what you do, the, the trust thing and once they get to trust you and once they feel easy and comfortable they become more compliant with information that you’re gonna give ‘em, they’re more open to let you have a look at them, you know, the injury ‘cause obviously their first thing is, is “this is gonna hurt if I let him touch it” … So the big softly softly approach first before anything else really.’*

Participant P09

Limited scope of analgesics was identified as a barrier to effective pain management for children, with participants noting the lack of intermediate analgesic options:

*‘or if there’s some alternative some intermediate or, because we literally do go from ibuprofen, Calpol® to morphine, with zero in between’*

Participant P08

### Proposed improvements

**Fig. 2:**
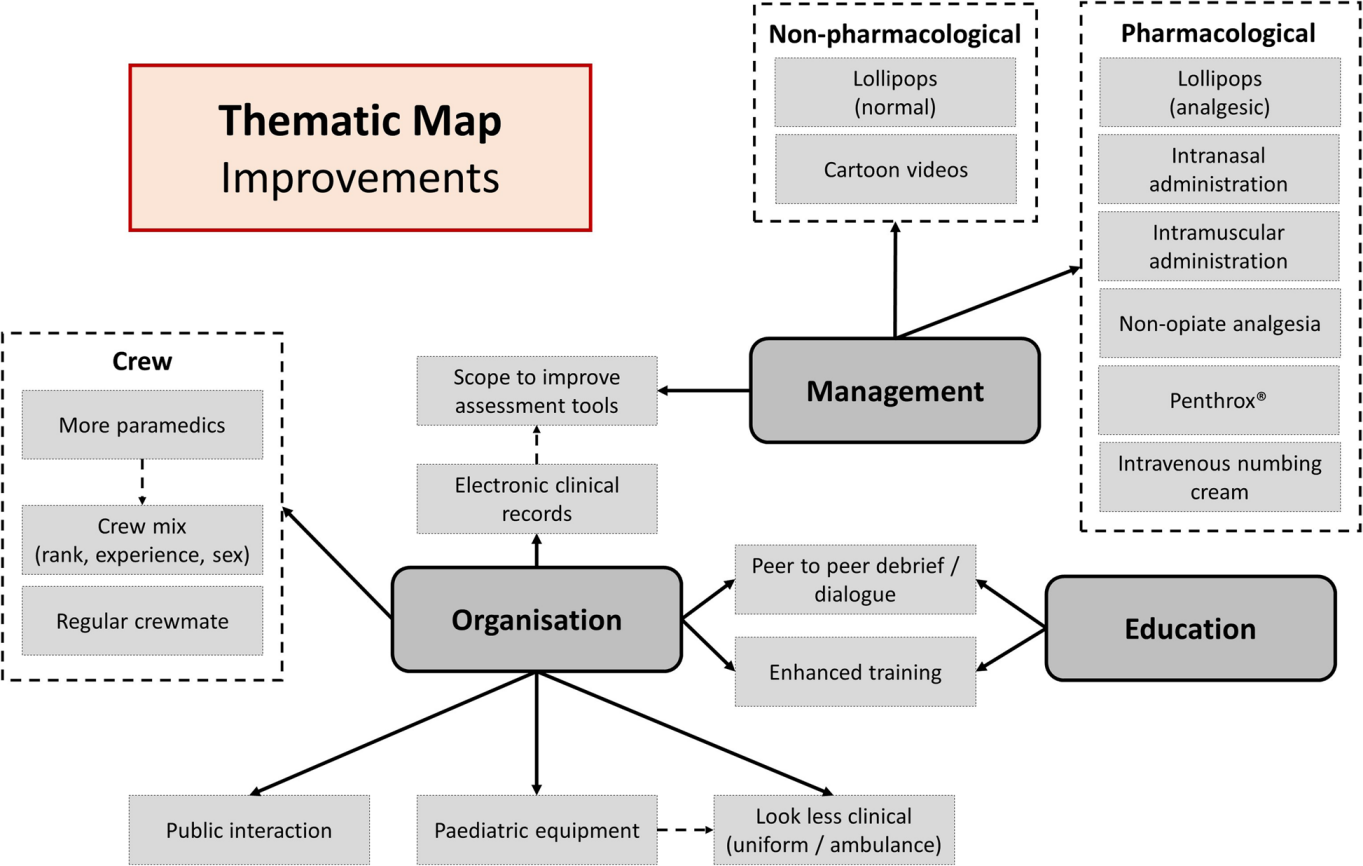
Thematic map of proposed improvements

A thematic map was generated to illustrate the proposed improvements, suggested by ambulance clinician participants, see Fig. 2.

Improvements revolved around three major themes; management, organisation and education. These major themes were informed by sub-themes, which were informed by direct quotations, see Additional file 1 for the complete table of supporting quotations.

#### Supporting quotations

Participants identified the intranasal route of drug administration as potentially beneficial, as they felt it provided a rapid and pain free method:

*‘the method of administration [intranasal] is much kinder for a child, the quick squirt up a nostril, is, is universally acceptable isn’t it, to all children ages … It’s not painful … its onset is really quick isn’t it … And that’s what you want with a young child isn’t it, you want them out of pain quickly.’*

Participant P03

Optimisation of the crew mix was discussed during the interviews, with participants stating that male and female clinicians might approach children in pain differently, arguing for a mixed sex crew:

*‘Whereas erm, the male erm, clinicians tend to go down assessment and management, at different routes, at different times, so, men tend to assess and manage and then try and soothe and comfort whereas females try and do the other, you know, spend more time on the comforting and soothing.’*

Participant P04

Participants stated that ambulance services could make more effort to look less clinical, perhaps by altering the inside appearance of the ambulance or altering staff uniform, with one participant proposing child friendly tabards:

*‘there's not fluffy teddy bear or a paediatric shirt in sight, whereas we know paed nurses generally are a bit fluffier with teddy bears or tabards or something that made them cuddlier, whereas we’re just not [laughter] … I’d love to see ambulance staff with teddy bear [laughter] teddy bear tabards.’*

Participant P08

### Driver diagram

**Fig. 3:**
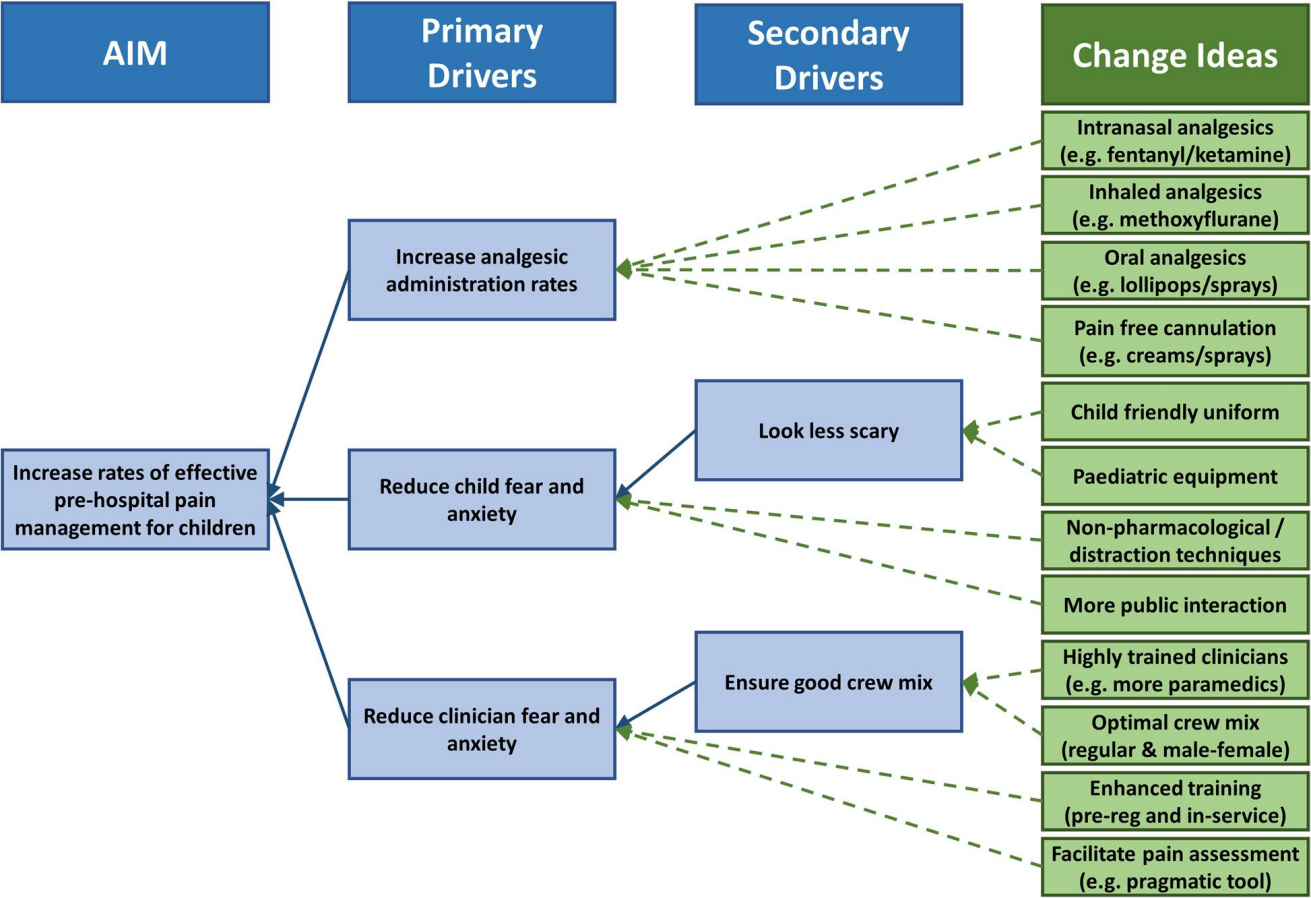
Driver diagram

The barriers and facilitators illustrated in Fig. 1 along with the proposed improvements illustrated in Fig. 2 were combined with the findings of our previous systematic review [12] and mixed methods study [2, 21] to generate a driver diagram, [29] see Fig. 3.

The primary drivers, illustrated in Fig. 3, show that rates of effective pre-hospital pain management in children may be improved by increasing rates of analgesic administration and reducing child and clinician fear and anxiety.

## Discussion

Paramedics and emergency medical technicians (EMTs) identified several barriers and facilitators along with proposed methods of improvement. These were combined with previous evidence to develop a plan to improve the quality of care for children suffering acute pain in the pre-hospital setting.

### Barriers and facilitators

Many of the identified barriers and facilitators within the management, emotional, social, organisational and knowledge and experience themes had previously been identified [14, 30–33]. Some were considered novel in this population and setting; physical (visualisation of trauma), child shame, child embarrassment, clinician empathy, the child’s prior experience of pain, the clinician’s life experience, service demand and environmental factors (light, noise and colour).

Shame experienced by children, particularly adolescent children, has been studied in sport, where adolescent athletes have a fear of failure and subsequently a fear of shame and embarrassment [34]. Shame typically leads individuals to hide, deny or escape interpersonal interaction [35]. This may explain why shame was identified as a barrier, as children may be less likely to interact fully with the clinician and perhaps less likely to truthfully report pain. Shame may also explain why male children are more likely to achieve effective pain management than female children in the pre-hospital setting, [12] as our previous study suggested male children may display more ‘bravado’ [21], thus skewing pain scores.

Clinician empathy was identified as an influencing factor. Patients attended by clinicians with high levels of empathy are significantly more likely to have reduced severity and duration of illness [36] and are more likely to retain information and comply with self-administration of medication [37]. Maintaining empathy can be difficult; the findings of this study highlighted a number of factors that can influence the clinician’s level of empathy, including health status, run of shifts, job types, how busy the clinician has been and the time of day or night.

Environmental factors such as light and sound were identified as influencing factors, but evidence to support this was sparse. The physiological impact of light on pain is not clear. One study showed that supplementary bright light and even low light was effective at reducing pain intensity in adults suffering nonspecific back pain [38]. Animal studies have shown green light to be a promising mechanism to promote antinociception [39]. The optimum brightness and colour of light to promote effective pain management is currently unknown and requires more research. Audio-analgesia, the use of sound to suppress pain, has also been described extensively, [40, 41] with white noise being used to soothe new-born babies for example [42]. The impact of noise from crowding or from the ambulance or medical equipment on pain perception is less clear and requires further research.

### Proposed improvements

Some of the proposed methods for improvement had previously been identified, such as use of intranasal analgesics, topical numbing creams, enhanced education and enhanced pain assessment [14, 30–32]. Several were considered novel; lollipops, analgesic lollipops, cartoon videos, methoxyflurane (Penthrox®), non-opiate analgesics, and electronic clinical records (to facilitate pain assessment).

The interaction between sugar and pain in children has been researched extensively; the effectiveness of sugar for treating pain in neonates was confirmed in a recent systematic review [43]. Oral glucose was effective at reducing distress in infants up to the age of 12 months [44]. Intraoral sucrose may be effective at reducing pain in pre-pubescent children [45] and sucrose was effective at increasing the pain threshold and tolerance in children aged 5–10 years [46]. The combination of an active analgesic agent (fentanyl) coupled with the sugar (sucrose) of a lollipop is therefore an appealing intervention. In the acute setting, emergency department studies showed that fentanyl lollipops were effective in children at reducing pain [47, 48] and two battlefield studies [49, 50] concluded that oral transmucosal fentanyl citrate provided rapid non-invasive analgesia that was safe and effective in injured army casualties. Further research is required to determine the safety and efficacy of oral transmucosal fentanyl citrate for children in the pre-hospital setting.

Methoxyflurane has been utilised in Australia for pre-hospital child pain management for many years [51] and is currently undergoing a clinical trial within the UK to determine efficacy and safety in children [52]. Methoxyflurane may have the potential to replace nitrous oxide (Entonox®) as the pre-hospital inhaled analgesic of choice within the UK as it is less cumbersome and more child friendly.

Topical creams are effective at reducing pain in children during needle insertion [53]. The concern for pre-hospital use is the delay in action. When a child is suffering acute illness or injury, rapid interventions are necessary to reduce suffering and facilitate extrication and transport to hospital. Consideration should also be given to onward care; it would be useful for the hospital to be able to cannulate the child after arrival if further analgesics or other drugs are required.

Optimum crew mix was discussed by participants; having a regular crewmate was considered important, with many stating that it makes difficult cases easier to manage as clinicians are familiar with each other and their working practices are well rehearsed, so they can focus on the patient. One study found that having a regular crewmate enhanced the psychological coping strategies of critical care paramedics when dealing with life threatening events [54]. This may make coping with similarly stressful situations, such as managing a child with severe acute pain, easier. Clinician sex mix was also deemed important by participants, as male and female colleagues may have differing approaches. Children and parents may have differing views on the sex of the attending clinicians; Waseem and Ryan [55] studied 200 children (70% male) aged 8 to 13 years attending a paediatric emergency department for laceration repair. They found that 79% of children who needed a suture in the emergency department would prefer to be treated by a female doctor (whereas 60% of parents preferred a male doctor). Jepsen and Rooth et al [13] found that parents of children attended by ambulance perceived female clinicians to provide more confident and more natural care for their children.

The combination of having a crew of clinicians who work together regularly, who are of opposite sex and contain at least one paramedic could improve rates of effective pain management in children suffering acute pain. Further research exploring the perceptions of children and parents would be ideal to help develop a theory for the ‘optimum crew’ mix.

### Driver diagram

The driver diagram, illustrated in Fig. 3, showed that rates of effective pain management may be enhanced by increasing rates of analgesic administration and reducing child and clinician fear and anxiety. Inhaled (e.g. methoxyflurane) and oral (e.g. lollipops) routes have been discussed above. Studies have shown that the introduction of intranasal fentanyl improves the rates of effective pain management in children suffering acute pain in the pre-hospital setting [56, 57]. A recent rapid evidence review found that intranasal fentanyl appeared to be effective and safe, but interventional data were lacking [58]. Clinical trials are needed to corroborate this finding.

Reducing fear and anxiety in children could be achieved through child friendly uniform. Whilst theoretically this is feasible, the practicalities need consideration due to infection prevention and control concerns and it would have to be a temporary item of clothing that could be donned and removed for appropriate incidents only (a tabard perhaps). Paediatric nursing staff have altered their clothing to improve the experience of children for many years, with brightly coloured uniforms preferred by children [59–62] which reduce anxiety [63, 64] and increase positive emotions, for example feeling calm, relaxed or happy [61]. There is potential for similar benefits in the pre-hospital setting.

Children attended by paramedics are more likely to achieve effective pain management than those attended by EMTs [12]. Optimum crew mix was discussed by participants; ensuring a paramedic, or highly qualified clinician, is on each emergency vehicle may help to reduce the overall fear and anxiety of the crew, as paramedics were perceived to be more confident, more experienced and have an extended scope of practice [21]. This necessitates long-term commitment to staff training and development by ambulance services.

### Strengths and limitations

Many of the findings from this study were previously identified, demonstrating external validity, therefore the cumulative recommendations illustrated in Fig. 3 may be transferrable to emergency medical service settings outside of the UK. Several novel barriers, facilitators and potential methods of improvement were identified within this study; this contributes to a more comprehensive understanding of this complex phenomenon and could help to improve the quality of care.

The low number of EMT participants could be perceived as a limitation, however we felt that code and meaning saturation were achieved and that further EMT data were unlikely to provide any new insights. Due to the clinical background of the interviewer, ‘blind spots’ were a concern, [24] where seemingly simple concepts that are taken for granted may have been overlooked. Involvement of a non-clinician (GRL) and clinicians from different clinical fields including nursing (PH) and primary care (ANS), along with review from a paramedic researcher in a different ambulance service (CW – acknowledged) helped to minimise the impact of these blind spots on the analysis and interpretation.

### Implications for policy, practice and research

National level initiatives that encourage the measurement of pain, strengthening the audit of pain assessment in children, should be introduced. Knowledge mobilisation strategies should be implemented within ambulance services to reduce the gap between research and clinical practice.

Clinical practice recommendations include increasing rates of analgesic administration, by utilising different analgesics and routes. Where efficacy and safety data are lacking, then clinical trials should be performed. Reducing the fear and anxiety experienced by children during emergency callouts could be achieved via child friendly uniforms, enhanced non-pharmacological distraction techniques, utilising more paediatric equipment and participating in more public interaction. Increased public interaction would allow children the opportunity to familiarise themselves with the ambulance staff, vehicle and equipment and could be achieved through attendance to schools, public events or holiday venues. Reducing the fear and anxiety experienced by clinicians could be achieved by optimising the crew mix, by having a paramedic (or highly qualified clinician) on all vehicles, ensuring male and female crews where possible and allowing crews to work together on a regular basis. Pragmatic pain assessment tools for children in the pre-hospital setting should be explored, developed and implemented and paediatric training should be enhanced.

Future research should involve children and their parents to explore their experiences, determine the most important outcome measures and co-produce interventions to improve the quality of care.

## Conclusion

Pre-hospital pain management in children is complex with biological, psychological and social factors to consider with the interactions between child, clinician and parent. Pain management may be improved by increasing rates of analgesic administration and reducing the fear and anxiety experienced by children and clinicians. Investment in future research and intervention development is imperative; we need to make pain matter [65]. Only then can we improve the quality of care we provide to children suffering acute pain in the pre-hospital setting.

## Supplementary Information

**Additional file 1.**

## Data Availability

The datasets generated and analysed during the current study are not publicly available due to the ethical approval parameters, but are available from the corresponding author on reasonable request. Supporting quotations are included in the supplementary file.
