# Supplementary material for: Improving ambulance care for children suffering acute pain: a qualitative interview study
Source: BMC Emerg Med. 2022 Jun 3;22:96. doi: 10.1186/s12873-022-00648-y (PMC9164349; doi:10.1186/s12873-022-00648-y)
Supplement: Supplementary file 1 — Additional file 1. [file 12873_2022_648_MOESM1_ESM.docx]

**Additional file 1**

Improving ambulance care for children suffering acute pain: A qualitative interview study

Table of Contents

[Appendix 1. Interview schedule. 2](#_Toc94261227)

[Appendix 2. Participant characteristics. 3](#_Toc94261228)

[Appendix 3. Quotations to support the barriers and facilitators. 4](#_Toc94261229)

[Appendix 4. Quotations to support the proposed improvements. 17](#_Toc94261230)

**NB.** The data relating to section 2 of the interview schedule (see Appendix 1), seeking explanations for disparity in care between groups, has been published separately: Whitley, GA, Hemingway, P, Law, GR, Siriwardena, AN. Ambulance clinician perspectives of disparity in prehospital child pain management: A mixed methods study. Health Sci Rep. 2021; 4:e261. <https://doi.org/10.1002/hsr2.261>

# Appendix 1. Interview schedule.

**Date: Participant ID Number:**

**Pre-Interview:** PIS & Privacy Notice Read? □ | Questions? □ | Consent Form □

1. Can you tell me about a time you have managed acute pain in a child under 18 years?
   1. What made the process more difficult? Or easier?
2. Can you think of any groups of children that might receive more effective or less effective pain management?

| **Child Age □** | **Paramedic Crew □** | **Analgesia Administration □** | **Deprivation □** | Child Sex □ |
| --- | --- | --- | --- | --- |
| Child Ethnicity □ | Trauma / Medical □ | Distance to Hospital □ | Treatment Administration □ | Clinician Experience □ |
| Clinician Sex □ | Clinician Age □ | Clinician Ethnicity □ | Clinician Status as Parent □ | Other |

Other:

1. What are the barriers and facilitators to managing pain effectively in children?

| Fear □ | Education & Training □ | Experience / Exposure □ | Colleagues □ |
| --- | --- | --- | --- |
| Relatives □ | Assessment □ | Management □ | Experience of the Child □ |

Other:

1. How could pain management for children be improved in the future?
2. Any Questions?
3. Comments:

# Appendix 2. Participant characteristics.

| Characteristic |  |
| --- | --- |
| Age, years |  |
| Median, (IQR) | 43.5 (41.5, 45.75) |
| Mean, (SD) | 42.33 (6.02) |
| Minimum value | 30 |
| Maximum value | 49 |
| Sex |  |
| Male, n (%) | 7 (58) |
| Female, n (%) | 5 (42) |
| Rank |  |
| Paramedic, n (%) | 9 (75) |
| Emergency Medical Technician, n (%) | 3 (25) |
| Experience, years |  |
| Median, (IQR) | 12 (4.25, 15.5) |
| Mean, (SD) | 10.75 |
| Minimum value | 1 |
| Maximum value | 23 |
| Parent status |  |
| Yes, n (%) | 7 (58) |
| No, n (%) | 5 (42) |

IQR – interquartile range, SD – standard deviation

# Appendix 3. Quotations to support the barriers and facilitators.

| **Quotation Number** | **Quotation** | **Sub-theme** | **Theme** |
| --- | --- | --- | --- |
| 1 | ‘if they can see it erm, it’s, it’s not normal erm, they’re gonna, they’re gonna erm potentially exacerbate the, it’s, they’re gonna exacerbate their own erm, distress aren’t they really, erm, if a, if a child can visually see that their ankle’s pointing the other way, erm, it’s, that’s, that’s not gonna be good for them, they’re gonna feel, they’re go-, they are gonna feel that pain quite a lot, especially when we start sort of manoeuvring and manipulating or whatever … Erm, if there’s blood, obviously, erm, children resemble blood as bad so you know they, that sort of makes things worse, erm, so yeah I think erm, the, the visualisation of it’  Participant T01 | Visualisation of trauma | Physical (bodily sensations) |
| 2 | ‘Yeah, yeah, they, they can see that something has changed on their body as opposed to something that’s, inside the torso … That they can’t, that they have no idea what, what’s causing it. That, that could equally be as, as traumatic but, I think v-visualising something can be as bad if not worse…’  Participant P01 |  |  |
| 3 | ‘I think it’s very difficult to distinguish what is fear and what is pain, and we could end up highly scoring a child for pain, because they’re hysterical, and saying they’re a 10 out of 10 pain and we could end up over-treating… because maybe we’re treating fear’  Participant P03 | Child fear/hysteria | Emotional (mental sensations) |
| 4 | ‘once we’d got the initial hysteria and anxiety controlled, that erm, allows to, to break down that, that cycle that, that erm inward cycle of, of, of pain.’  Participant P04 |  |  |
| 5 | ‘And it was just [click] like flicking a switch, once he calmed down that was it, we got something into him and explained everything, he was fine but it’s just trying to break down them initial barriers.’  Participant P06 |  |  |
| 6 | ‘Erm, I think then they’re starting to get more body conscious, erm so they might not want you to strip them down … he refused take any of his clothes off because he was embarrassed … Erm, and I guess that’s not logical it’s, but at that age you’re probably not as logical as you are when you’re, through the joys of adolescence erm, so yeah I think it gives you a whole different set of challenges when they’re starting to get pre-pubescent and teenagery’  Participant P08 | Child embarrassment |  |
| 7 | ‘I think little boys sometimes, are fearful that they let their dads down … By showing pain, or crying … Or wimping’  Participant P07 | Child shame |  |
| 8 | ‘I think people are scared of giving pain relief to children, erm, more advanced pain relief especially because of the effects it may have, and it’s not something we do very often so it’s quite a big thing to think about, and then gaining access to give further pain relief, Calpol is one that you know, people give to their own children and, but anything more than that is quite a, it’s a barrier for the clinician I think.’  Participant P05 | Clinicians fear treating children |  |
| 9 | ‘I think, historically for ambulance staff, I think if you ask any, any ambulance member of staff what they dislike going to most it’ll either be maternity or children, and it’s because of the complexities of children … I’ve worked with a few people in the past that’ll just go “oh my god it’s a kid, can you deal with it?”’  Participant P09 |  |  |
| 10 | ‘P: Yeah I think it's times in life erm, and I guess that’s around you as a person, what you're going through with life at the moment, how well you are, how, busy you are, the run of shifts you’ve had recently, the jobs you’ve had, whether you’re happy, healthy.  I: Do you think that could influence your assessment and management of children?  P: Yeah erm, and again that’s that kind of burn out you know if you get into the clinician burnout fatigue thing you're not gonna manage any job as well as you should be doing’  Participant P08 (P=participant, I=interviewer) | Clinician empathy |  |
| 11 | ‘So, middle of night, people are tired, that always seems to have, although it should never do, it always seems to have an effect on how people erm, approach people or some individuals approach people’  Participant P09 |  |  |
| 12 | ‘Because it’s not just a case of managing the child is it, you’re managing the parents as well, because quite often, they, they could exacerbate the situation, you know if they’re really stressed or really distressed, that feeds into the child as well, erm, so you’re trying to manage both the child and the parent at the same time, erm, and I think if you can calm both down, you know, “I’m not panicking, no need for you to panic” you know, then, that helps the situation’  Participant T03 | Importance of managing parents | Social (interpersonal, societal and family relationships) |
| 13 | ‘And then you have other parents where, they’re almost so, so laid back they’re horizontal, “oh yeah they’ll be fine”, and the situation’s a lot easier to cope with, they’ll be more cooperative, they’ll help with what’s needed, they’re quite pragmatic and very, you know, “sorry we’ve had to call you but we couldn’t get them in the car” or “this has happened” or… you know. And you can see they’re panicking underneath but they’re keeping it calm for the child so that it does have a big, yeah, it does have a big influence on what, what’s happening, in the attitude of the child, because that’s how children learn isn’t it, it’s from parents and the reaction of the people around them, and that’s how they learn to react to certain events.’  Participant P02 |  |  |
| 14 | ‘dad was vomiting, so dad had lifted the trou… erm, pulled the sock down and he’d seen this fracture and dad was vomiting and that terrified the child as much as the injury that the child had. I think if the parents are really stressed, then the child picks up on that stress, whereas I think calm parents help calm children.’  Participant P03 |  |  |
| 15 | ‘if the parents are frantic and you can’t get through to a parent there’s no way you, the child’s not gonna trust you either.’  Participant P05 |  |  |
| 16 | ‘Yeah so, gaining trust from a child is erm, quite difficult so, erm, she was not happy, she was cold, that was her biggest problem because she’d been put in a shower, erm, so getting anywhere near a child that is cold is quite difficult especially when they don’t know you so, getting the burns dressings on as quick as possible so that she could then have something over the top of her skin, her favourite jumper, which then trusted us, she then trusted us and she was quite happy then, then to take pain relief, if we hadn’t have done that first there was no way that she would have had anything from us, any observations.’  Participant P05 | Develop trust with child |  |
| 17 | ‘The erm, the interaction with the child, I think gaining the trust erm, is a massive thing, it, once you’ve lost that with a child, sometimes it doesn’t really matter what you do, the, the trust thing and once they get to trust you and once they feel easy and comfortable they become more compliant with information that you’re gonna give ‘em, they’re more open to let you have a look at them, you know, the injury ‘cause obviously their first thing is, is “this is gonna hurt if I let him touch it” … So the big softly softly approach first before anything else really.’  Participant P09 |  |  |
| 18 | ‘if you try and go in all guns blazing and “right we need to do this and we need to do this” and there’s some of them that, they get this tsunami of, of strangers coming in, interfering with them and trying to put things on them and, and that’s upsetting itself as it is … So it’s, it’s being a bit softly, softly and coming in and… letting the child get used to you being there before anything happens, because it’s already, they’re already in a bad place anyway…’  Participant P01 | Calm relaxed approach |  |
| 19 | ‘Like you say, it’s a matter of calming them down isn’t it initially and, trying to get the, because when you go in, that whole situation is quite heightened isn’t it, but it’s very different after about 10 minutes when you’ve built up a bit of a rapport and you’ve got them a little bit quieter and calmer you can probably get a, a truer sense of, of what’s happening and how their feeling, as opposed to when you’re going in and they’re screaming and crying initially’  Participant T03 |  |  |
| 20 | ‘my crewmate is my teammate, I’m assessing child, my crewmate is reassuring parents, I’m assessing child or getting things ready, erm, my crewmate is entertaining child’  Participant P04 | Teamwork with colleagues |  |
| 21 | ‘trying to get him settled down, which actually my crewmate did a better job than I did, erm, crewmate was being erm, female, so I think him being a young lad found that more comforting, erm, I wouldn’t say, I use the word “mumsy” but she’s got a child of her own so she’s a lot more switched on, so she had that sort of empathy there’  Participant P06 |  |  |
| 22 | ‘I think that crews should be mixed crews, as, as much as you can do, erm, for the reason that different patients respond better to different sexes’  Participant T03 |  |  |
| 23 | ‘certainly seen people hinder the process and that’s just like going in and being very loud and erm, insensitive to the situation erm, only speaking to the parents and then expecting a child to agree to have all these assessments done, erm, not getting down to their level and not making them the forefront of what you’re doing…’  Participant P05 |  |  |
| 24 | ‘Erm, and not very often is there, when I attended a patient now, erm, is there a vehicle available in a short period of time, to take them to hospital, very often … the demand outstre-, erm outstrips the, the resources, so therefore I have to think about how I'm going to manage them in the meantime’  Participant P07 | Service demand | Organisational |
| 25 | ‘with the, the child, child under two policy for example, erm, clinicians are very quick to just go “well they’re gonna go to hospital anyway because policy says they need to go to see a Doctor”’  Participant T01 | Policy |  |
| 26 | ‘but with the aid of such things as Pathfinder [clinical decision aid], erm, that does make things, it gives you the guidelines and the framework to make, to make good decisions and minimise erm, disagreements between clinicians, I’d say, because we’re all here to, we all do this job, we all understand that that’s, you know, we have those, those frameworks in place to, to help us and to help the patient’  Participant T02 |  |  |
| 27 | ‘A lot of the kit that we’ve got, things like the traction splints and things are all set up, for adults, there’s no paediatric versions of some of the stuff that we carry…’  Participant P02 | Lack of paediatric equipment |  |
| 28 | ‘We don’t have paediatric mouth pieces for Entonox®, the alternative is we put a mask over their face and we help deliver it, and that’s not, that’s not nice either is it.’  Participant P03 |  |  |
| 29 | ‘Erm, we don’t, I’m trying to think I’m honestly trying to think the last time we had any sort of child training … and I can’t remember when it last was…’  Participant P02 | Limited service education and training |  |
| 30 | ‘If you look like at our ‘Stat and Mand’ [statutory and mandatory training] we don’t do anything on paediatrics, unless it’s cardiac arrest, that's what we focus on’  Participant P08 |  |  |
| 31 | ‘When you first qualified, if you’ve got a 5 minute journey to hospital and you’re not sure how to deal with something, you scoop them and take them to hospital, and you almost make that somebody else’s problem, because they’re more experienced they could put the cannula in easier they could choose the pain relief, you’re a bit nervous about your options with children.’  Participant P03 | Distance to hospital |  |
| 32 | ‘Yeah, I think children who are closer to hospital erm, they’ll, I think that, the, the simple erm, oral an-, oral paracetamol, erm, that can go down, because we can get that down easily, maybe ramp up quickly to, to an Entonox® so they are getting analgesia, great, but if they needed anything else, if they needed to go to that next level the, the top level of analgesia, not a chance are they getting it, not at 5 minutes out, we’ll get them to hospital every time.’  Participant P04 |  |  |
| 33 | ‘yeah if you’re further away and, and there’s still pain evident, you have to, you have to do something, because that’s, that’s our job [laughter] if we don’t, if we don’t try and address the pain we’re not really doing our job, erm, which is problematic, I think.’  Participant P01 |  |  |
| 34 | ‘whether you’re 20 miles away or 2 miles away, I wouldn’t, I wouldn’t withhold pain just because I was close to hospital … patient comes first rather than distances, times, protocols, whatever, the presentation of the patient is what is important and making them comfortable, the distance isn’t a factor, is not a factor to me.’  Participant P09 |  |  |
| 35 | ‘and the music was enough to erm, to soothe him, erm, and I liked it in the back of the ambulance so I put like the dim lights on or put the blue light on or, something that’s a bit different to just to, a bit of distraction.’  Participant T03 | Light (dim light helps) | Environmental (external background of human experience) |
| 36 | ‘Straight off is erm, noise, distraction around, so I try and calm the situation’  Participant P04 | Noise (from nearby people and ambulance) |  |
| 37 | ‘they just don't know what this big yellow thing does with all these noisy lights and this equipment and you two are just strangers and “who the hell are you?” and you've got all these big bulky pockets’  Participant P08 |  |  |
| 38 | ‘you know we’re not friendly looking, big green uniform and a big yellow ambulance they’re like “oh my god, I’m gonna be taken away in that, they’re gonna take me away from my parents”’  Participant P08 | Colour (scary uniform and clinical ambulance) |  |
| 39 | ‘Reluctancy for the child to want you to do anything because of fear, so the jolly green giant walking through the door’  Participant P09 |  |  |
| 40 | ‘in, younger children, I can’t remember what the age is now where pain pathways develop properly in children, but certainly in younger children they can’t always locate pain very well…’  Participant P02 | Pain assessment is challenging | Management |
| 41 | ‘we’ve got the Wong & Baker FACES®, which come with a very long detailed explanation if you do it properly, and an explanation that is not suitable for a, certainly not suitable for a pre-school child, but it’s complicated even at 7, 8, 9, the explanation is far too complicated’  Participant P03 |  |  |
| 42 | ‘I tend to just put a description on you know, child very unsettled, erm, looks miserable, withdrawn, screaming what, whatever the reaction is just to try and give a snapshot on the patient report form, what that child’s like when I got there…’  Participant P09 |  |  |
| 43 | ‘If, if it’s someone, if it’s a child or sort of, who’s sort of not verbalising quite as much then I’ll do it off my perception I guess with the Wong scale … Erm, so if they’re crying I’d work well they’re 9, 10 up here aren’t they … I’m then using my perception of it a little bit more than their perception, erm, it’s sort of that, that sort of quite difficult age where you can’t really get a definitive number so you’re really yours, your, your opinion on it really’  Participant T01 |  |  |
| 44 | ‘but Entonox®, the delivery systems, like, you have to stop a child crying, because they have to stop crying to actually take Entonox®, you can’t cry and suck at the same time, so the child needs to be calmed, and calming a child who’s in agony, and getting them to coordinate their breathing to suck, is like trying to talk a hyperventilation in the peak moment into, into breathing slowly isn’t it … and I think it’s, it’s big and it’s cumbersome and it’s you know it’s a big cylinder and it makes a noise and the mouth pieces are quite big, and the masks are quite scary, so the delivery system is not really tailored for children.’  Participant P03 | Entonox difficulties |  |
| 45 | ‘I’m always very aware with those sorts of jobs when a child’s in a lot of pain like that, that the, paracetamol and the Nurofen® take some time to kick in, and when you’ve got children it’s very hard to explain that they’re gonna have to wait, for that pain relief to kick in, so there’s nothing much that I can give a child…’  Participant P02 | Oral analgesics are slow |  |
| 46 | ‘I just think our pain relief management for children is not, not as easy for children as it is for, they’re used to swallowing medicine aren’t they? But it doesn’t work quick enough in trauma.’  Participant P03 |  |  |
| 47 | ‘But certainly age would have done, because my consideration if they’d been 3, and they’ve got say puppy fat on the back of their hands still … The cannula’s harder to go in, the ACFs [antecubital fossa] not a great place for a young child, because they, you stop them bending their arms, the choice of sites is, the choice of sites is more limited the veins that you can find are more limited, the child moving and, and not understanding to stay still because they’re so young and their comprehension of staying still and not, they’re more likely to fight you aren’t they, the younger the child the more likely they are to pull away.’  Participant P03 | Cannulation is painful and difficult |  |
| 48 | ‘I thought cannulation would exacerbate her, her level of pain, her level of anxiety, so I went for a, an oral route rather than, than for vascular access.’  Participant P04 |  |  |
| 49 | ‘I think it's terrifying because we don't cannulate children that often … you don't want to keep repeating it because you know you're gonna hurt them and cause more fear and terror and, that sort of thing of erm, gonna make them phobic for the rest of their life sort of thing 'cause you’ve fumbled about’  Participant P08 |  |  |
| 50 | ‘to my mind the paracetamol and the Nurofen® are at the bottom of the pain, ladder if you like for treatment, and then I’ve got Oramorph right at the top and there’s not an awful lot in between the two to help manage the pain, but, and nothing that acts particularly quickly’  Participant P02 | Limited scope of analgesics |  |
| 51 | ‘or if there’s some alternative some intermediate or, because we literally do go from ibuprofen, Calpol® to morphine, with zero in between’  Participant P08 |  |  |
| 52 | ‘things like distraction and using other bits of kit can help as much as giving the pain relief, I think we get very focussed on, what analgesics we’ve got to use without remembering that there’s other things you can do to help pain management’  Participant P02 | Non-pharmacological techniques are helpful |  |
| 53 | ‘I think all children have a level of pain management we can erm, address, non-pharmacologically.’  Participant P04 |  |  |
| 54 | ‘I think with the younger kids you’ve got a very narrow gap of their perception of pain, an older child, same as adults you know, life experience up to that point has dealt them, so, various amounts of pain, so from their point of view they’ve got a, a better understanding of how bad that pain is, to a certain degree’  Participant P06 | Child’s prior experience of pain | Knowledge and experience |
| 55 | ‘The other thing is as well, children that are on long-term treatment are more resilient and will definitely allow you to do nearly anything that you ask them to do, so oncology patients they’ll, kids, there’s not many of them that’ll say “oh I don’t want a cannula”, because they’ve had that many, they’re quite used to it.’  Participant P09 |  |  |
| 56 | ‘I don’t think there’s enough training on pain management in children in general’  Participant P06 | Education and training |  |
| 57 | ‘We didn’t have a lot of time… erm, in training school, and I think nothing prepares you for being out there on the road does it? Nothing, nothing prepares you for a screaming child, erm… particularly you know, when you’ve not been exposed to it very much erm, you know, and I suppose and also, particularly the trauma jobs you know nothing exposes you to, to seeing a child with a horrific injury erm, so no I don’t think you’re ever prepared for it really.’  Participant T03 |  |  |
| 58 | ‘We’re very much told, or taught at an early stage, that, you may, I’m sure you will have heard of this before but, babies and children compensate, compensate and then fall off a cliff, you know then they, they, when they no longer compensate, it’s almost as though training college, you were taught to consider children as a ticking time bomb, that even a well child will suddenly become very unwell and I think we, we set ourselves up very much for a fall’  Participant P04 |  |  |
| 59 | ‘It gave me the skills to assess children and get a rough idea what’s going on if it’s something acute’  Participant T02 |  |  |
| 60 | ‘Yeah well the… It’s a, it’s quite a bit at the minute it’s a 2 year degree, whereas previously it was a 12 week in house erm, learn the management and, and go and that’s how it was until about 5 years ago with technicians as well until they’ve changed it and then they’ve all gone through the university route, so I think there’s been a massive change in the last 5 years.’  Participant P05 |  |  |
| 61 | ‘I think it’s on erm, as an inherent responsibility as a clinician, when we sign to HCPC we say that, one of our standards is to maintain our, our knowledge, maintain our CPD, I don’t think we get it to start off with, that doesn’t mean to say that we don’t look for it elsewhere, and there are some fantastic sites available’  Participant P04 |  |  |
| 62 | ‘I think, yeah, I think so, I think when you’ve got your own children and they’ve been in a lot of pain, it does change your management slightly and it changes the urgency with which you want to do things it’s very hard, erm … some of the things they’ve thrown at me over the years I probably wouldn’t feel nearly as confident if I was sat here at this age without my own children, there’d be a huge gap in my knowledge ‘  Participant P02 | Parent status |  |
| 63 | ‘from my personal experience I feel more confident dealing with children erm, young children because I have some my, of my own and it’s definitely empowered me to, be a bit more direct with my questioning, erm, and my erm, my management and my assessment.’  Participant T02 |  |  |
| 64 | ‘everybody that I speak to that’s a parent they said “I couldn’t imagine going to that, I wouldn’t know…”, because since having children they struggle to deal with other children, erm, that’s something that I hear quite often’  Participant P05 |  |  |
| 65 | ‘my youngest was, was really poorly … and I wondered how that was gonna react with me on the road if I saw a child in that similar condition and, funny enough actually erm, about two weeks after one episode at the hospital with my son it was, it was, which was quite distressing, we had a child very similar and all that sort of emotion went out the window and I just focussed on the job, so I didn’t, I didn’t treat it as a, I treated as a patient rather than a child, I just got on with it you know, this is what needed to be done, this is what I did and it wasn’t until afterwards where I reflected on with my crewmate thinking “actually, I reacted to that a lot better than I thought I was gonna do”’  Participant T01 |  |  |
| 66 | ‘Erm, it could be a disadvantage in some respects because, the clinician might think “why have they rung an ambulance? I deal with this myself”’  Participant T02 |  |  |
| 67 | ‘Erm, I think life experience would, would play into that, erm your approach, your line of questioning… and based on my experience, erm I keep going back to it but I think the older staff member would look at things holistically a bit more’  Participant T02 | Life experience |  |
| 68 | ‘I do think there is something to be said for life experience erm… and being exposed to certain situations through, through life experience and, some, some young people have been exposed to lots of different things but I think, I think that exposure, in life, puts you in, a, an advantage I think’  Participant T03 |  |  |
| 69 | ‘So you don’t get the exposure, which doesn’t build up your confidence base or your, your knowledge base … we learn by what we do you know and what we see, you know you can read your books, you can watch your webinars you know, “this is what a sick child looks like”, unless you’re sat there with it, and you hear them, you see them … That’s when it sinks in’  Participant P06 | Low exposure to children |  |
| 70 | ‘I just don’t think we deal with, it, it, because, because kids aren’t poorly, they’re never poorly are they? How many times to we go to children? As opposed, you know, if you looked over a year and you said that you’d been to 1000 jobs, how many of them would be children? I bet very, very, handful … And normally it’s, it’s febrile stuff isn’t it it’s, erm infection type stuff it’s … Temperature, it’s pyrexia, it’s never really pain it’s seldom we go to children with pain, so therefore erm… are we competent in dealing with child pain? No.’  Participant P09 |  |  |
| 71 | ‘we don’t necessarily deal with a lot of children that have had a lot of poly-trauma, so there’s that, lack of, knowledge, lack of experience, again the fear of doing the wrong thing…’  Participant P02 |  |  |

# Appendix 4. Quotations to support the proposed improvements.

| **Quotation Number** | **Quotation** | **Sub-theme** | **Theme** |
| --- | --- | --- | --- |
| 1 | ‘in every ambulance we have, we have little VDU [video display unit] screens, little video screens, why can’t we have some fun cartoons, I mean, there’s a concern that the erm, the crews maybe sat in the back at hospital watching Peppa Pig … Exactly, but, either those or, or tablets or something that we can erm, entertain, I know a lot of crews, a lot of parents now take their children’s Kindles or other tablets in with videos while they’re sat waiting in A&E waiting rooms, if you go into any one in the country you will see parents who’ve given up their mobile phones with videos’  Participant P04 | Cartoon videos | Management |
| 2 | ‘the method of administration [intranasal] is much kinder for a child, the quick squirt up a nostril, is, is universally acceptable isn’t it, to all children ages … It’s not painful … its onset is really quick isn’t it … And that’s what you want with a young child isn’t it, you want them out of pain quickly.’  Participant P03 | Intranasal administration |  |
| 3 | ‘it [Penthrox®] is easier, it's less scary than using erm a big blue gas cylinder in a big bag and a great big unwieldy pipe [Entonox®], which is really, really difficult for adults to even hold and manipulate and it will hurt your gums and your teeth if you twist it wrong and it, and that plastic thing smacks you in the mouth, you know, so maybe something like that, erm an appropriate size, erm, erm anaesthetist, erm anaesthetic gas it’s a really good way of doing it’  Participant P07 | Penthrox® |  |
| 4 | ‘if the potential is for that child to be cannulated, if we get to hospital and they’re still in pain for whatever reason i.e. they won’t accept the medicine or we can’t give it or whatever, at least if we could have got the Emla cream on then when they get to hospital the chances of them being cannulated is gonna be a lot quicker than them applying it and waiting half an hour for it to work, so would that be a better patient experience? Yeah. Would that be quicker pain relief? Yeah … Patient treatment would be a lot better … And more prompt, less suffering’  Participant P09 | Intravenous numbing cream |  |
| 5 | ‘if I’ve got something that was non-opiate based, that I could use that would be, that, that wouldn’t all, certainly they could give the diamorphine with it, that would be the best solution, for me.’  Participant P02 | Non-opiate analgesia |  |
| 6 | ‘Ketamine’s a buzz thing isn’t it at the minute, erm in pre-hospital care, and the fact that the side ef-, the long-term side effects of it is quite short, and that it’s given by erm somebody who’s gonna be very experienced, a very experienced erm paramedic or physician, that it’s some, that it, it, it would be a good thing to be, erm to be considered’  Participant P07 |  |  |
| 7 | ‘Or give us more confidence to do IM [intramuscular] injections if that's appropriate erm, just so that we fully understand the administration of morphine at smaller doses IM’  Participant P08 | Intramuscular administration |  |
| 8 | ‘but lollipops in younger kids are always a favourite, aren’t they, so, w-whether we actually need to be in the business of, of having some on the vehicle I don’t know, e-even if it’s a way of, of, of making that connection’  Participant P01 | Lollipops (normal) |  |
| 9 | ‘I: What kind of lollipops?  P: Is it fentanyl?  I: Yeah  P: Is it I think  I: So lollipops with an active drug?  P: Yeah active drug in ‘em yeah yeah … But obviously it’s an unbalanced [sigh] you know, the absorption rate’s gonna be slightly different, one lollipop doesn’t suit all’  Participant P06 (I=interviewer, P=participant) | Lollipops (analgesic) |  |
| 10 | ‘Wong-Baker, I’ve mentioned numerous times through this interview, I don’t think that’s always the, the best, version, it, it’s, it’s, there’s a lot of evidence for it but there are a lot of new versions, there are a lot of bright pretty coloured pictures, we have erm, erm electronic devices, erm, phones, we have erm, the Getac [electronic tablet], we can bring up, there’s a lot of different versions, with touch technology, so the, the child can, can, and audio, so child can point and, engage more, a little bit more fun perhaps, a little bit more attractive than a black and white, erm, [inaudible] face smiley’  Participant P04 | Scope to improve assessment tools |  |
| 11 | ‘but ultimately how we score the pain, I haven’t got an answer for it but I think we need a different system…’  Participant P06 |  |  |
| 12 | ‘a lot of these sort of big jobs we are having to at the moment be called, call, erm, have to call a paramedic in, erm, so potentially, erm, maybe mixing, having more para-tech crews’  Participant T01 | More paramedics | Organisation |
| 13 | ‘Whereas erm, the male erm, clinicians tend to go down assessment and management, at different routes, at different times, so, men tend to assess and manage and then try and soothe and comfort whereas female try and do the other, you know, spend more time on the comforting and soothing.’  Participant P04 | Crew mix (rank, experience, sex) |  |
| 14 | ‘And I think, when you first start you probably don’t have the confidence to take your time to assess, to try and solve the situation yourself, but I think that’s important that we do take time and we make the children feel comfortable and that we relieve the pain, because now, with experience I know that we can get to hospital and that child might still have to wait another hour in the corridor.’  Participant P03 |  |  |
| 15 | ‘I think that crews should be mixed crews, as, as much as you can do, erm, for the reason that different patients respond better to different sexes’  Participant T03 |  |  |
| 16 | ‘Human factors is huge, a huge issue erm, sometimes erm… erm some people you see might have a technician attending and a paramedic as the lead on the vehicle erm and they might very much work as a cohesive unit if they know each other well, but if they don’t know each other very well, there might be a little bit of awkwardness’  Participant P02 | Regular crewmate |  |
| 17 | ‘they say “oh put it in a splint this that the other”, they’re not the, the prettiest looking things, they look pretty medical and pretty scary’  Participant P06 | Paediatric equipment |  |
| 18 | ‘so let’s make it a bit more inviting, let’s make the, the inside of the ambulance look a little less, clinical, somehow, the, the touch of a button or, or there’s some pretty pictures erm, printed on the inside of the, the windows, that are normally covered by the blinds, but, I don’t know let, let’s just make ourselves look less scary to children.’  Participant P04 | Look less clinical (uniform/ambulance) |  |
| 19 | ‘there's not fluffy teddy bear or a paediatric shirt in sight, whereas we know paed nurses generally are a bit fluffier with teddy bears or tabards or something that made them cuddlier, whereas we’re just not [laughter] … I’d love to see ambulance staff with teddy bear [laughter] teddy bear tabards.’  Participant P08 |  |  |
| 20 | ‘Erm, so maybe, maybe this is where from a completely different aspect, we should be going more into schools and nurseries so that people aren't afraid of us, so that we take away, erm and I've done a couple of school visits to get kids on the back of an ambulance so that they’re not frightened of it, “so this is what this does, sit on the bed” or, you know “it goes up it goes down, it does this” erm, we can play with the kit a little bit, we can stick all the things on you, it doesn't hurt, erm, so then some of the child's fears gone away’  Participant P08 | Public interaction |  |
| 21 | ‘I think it’s better now we’ve got the Getacs I think, erm, I, I feel more confident getting a better idea of this, of that particular child’s pain, now.’  Participant T02 | Electronic clinical records |  |
| 22 | ‘possibly even more training and knowledge around things like straightening limbs or splinting… I don’t know, would be beneficial as well, I think there’s still quite a reluctance to, to move children too much, and to be fair a lot of the fractures are, are different in children where, things are bent rather than being, because of the sort of greenstick type of effect it’s, harder to know whether you can straighten or not, or what to do.’  Participant P02 | Enhanced training | Education |
| 23 | ‘But I also think we should be doing a lot more workshops on paediatrics in general, just to give people more confidence… can almost make it rolling CPD.’  Participant P08 |  |  |
| 24 | ‘when I say education I, I, I think it should be around about the peer group discussions … Erm, about keeping up-to-date erm, and knowledge, erm mentor-mentee discussions on things what went well and things what didn't go so well, because I think as a mentor, erm, I don't mind discussing things what didn't go so well, and it doesn't make me less of a mentor in fact I think it makes the mentee feel more comfortable to talk to me about things what they worry about and things that don't go so well, if they realise that I'm not infallible myself.’  Participant P07 | Peer to peer debrief / dialogue |  |
| 25 | ‘when I do my operational shifts it's nice that because I’ve possibly been quite lucky that I've had opportunity to do more and learn more and you can then pass that down the chain little bit, some people aren't quite doing things correctly, you can just tweak their practice a little bit, erm just discuss a few things’  Participant P08 |  |  |
